# Supplementary material for: Hot alkaline lysis gDNA extraction from formalin-fixed archival tissues
Source: PLoS One. 2024 Jan 2;19(1):e0296491. doi: 10.1371/journal.pone.0296491 (PMC10760679; doi:10.1371/journal.pone.0296491)
Supplement: S1 File — (PDF) [file pone.0296491.s001.pdf]

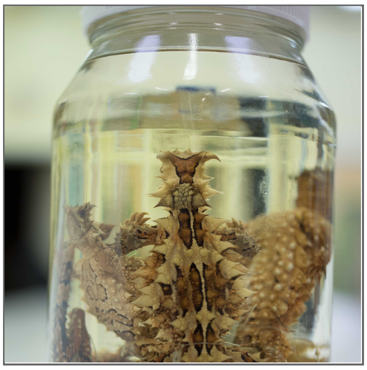

AUG 28, 2023

# Hot alkaline lysis gDNA extraction from formalin-fixed archival tissues

Erin E Hahn[1], marina.alexander[2], Alicia Grealy[1], clare.holleley[1]

[1]CSIRO National Research Collections Australia;
[2]CSIRO Health & Biosecurity

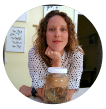

Erin E Hahn
CSIRO

OPEN ACCESS

**DOI:** dx.doi.org/10.17504/protocols.io.yxmvm32p6l3p/v1

**Protocol Citation:** Erin E Hahn, marina.alexander, Alicia Grealy, clare.holleley 2023. Hot alkaline lysis gDNA extraction from formalin-fixed archival tissues. **protocols.io** https://dx.doi.org/10.17504/protocols.io.yxmvm32p6l3p/v1

**MANUSCRIPT CITATION:** Hahn, E. E. *et al.* Unlocking inaccessible historical genomes preserved in formalin. *Mol. Ecol. Resour.* **22**, 2130–2147 (2022).

**License:** This is an open access protocol distributed under the terms of the Creative Commons Attribution License, which permits unrestricted use, distribution, and reproduction in any medium, provided the original author and source are credited

## ABSTRACT

The widespread practice of formalin preservation has historically limited genomic analysis of archival museum specimens.
Here we describe sample selection and extraction methods to recover genomic DNA sufficient to reconstruct complete mitochondrial genomes and achieve up to 20X nuclear genome coverage from formalin-preserved tissues.
We describe in detail our approach to vetting specimen preservation quality as well as our procedure for processing tissues with hot alkaline lysis (adapted from Campos et al. 2012) coupled with phenol-chloroform extraction and a small-fragment optimised SPRI bead clean up protocol.

## MATERIALS

### EQUIPMENT & CONSUMABLES

In addition to standard tips and tubes, you will need:

- pH meter that reads in ethanol
- Formaldehyde assay kit such as the Merck MQuant
- Covaris CryoPrep [or similar pulveriser]
- Nutator or other rocking platform
- Bench-top microcentrifuge (ideally one at 4°C and one at room temperature)
- Centrifuge with adapters for 15 mL tubes
- Autoclave
- Autoclave-safe screw cap 2 mL tubes
- Qubit, Tapestation, or similar method of quantifying low quantity dsDNA

### REAGENTS

**Ethanol dilutions to have on hand**

50%, 30% and 0% (water) chilled at 4°C

**Glycine Tris EDTA (GTE) Buffer**

Final - 100mM glycine, 10mM Tris-HCl, pH 8.0, 1mM EDTA

Store at 4°C

**Protocol status:** Working
We use this protocol and it's working

**Created:** Aug 08, 2023

**Last Modified:** Aug 28, 2023

**PROTOCOL integer ID:** 86084

**Keywords:** DNA extraction, formalin, genome sequencing, hot alkaline lysis, museum, formaldehyde

| Reagent | Volume for 50 mL |
| --- | --- |
| 10X TRIS Glycine Buffer (2M Glycine + 0.25 M Tris) | 2.5 mL |
| 0.5 M EDTA, pH 8.0 | 100 uL |
| ddH2O | Up to 50 mL |

**Alkaline Lysis Buffer**

Final - 0.1M NaOH, 1% SDS, pH 13

Store at room temp

| Reagent | Volume for 50 mL |
| --- | --- |
| 20 % SDS | 1.25 mL |
| 1N NaOH | 2.5 mL |
| ddH2O | Up to 50 mL |

**Small fragment-optimised bead solution (12% PEG/40% Isopropanol) preparation:**

- Pipette 1 mL of beads from stock into a 2 mL tube and wash 3x with ddH2O.
- In a 50 mL tube, add all reagents followed by beads resuspended in ddH2O.
- Store at 4°C.

NOTE: The PEG will not go into solution at this concentration. Mix very well before adding to DNA and it will dissolve once added. When ready to use, allow the beads to warm to room temp and then add 1.5X volume of bead mix to DNA.

| Reagent | Volume/ Mass |
| --- | --- |
| PEG-8000 for 12% in 83 mL | 10 g |
| 5 M NaCl | 10 mL |
| 1 M Tris-HCl, pH 8.0 | 500 uL |
| 0.5 M EDTA, pH 8.0 | 100 uL |
| Tween-20 | 27.5 uL |
| Washed Sera-Mag Beads | 1 mL |
| Isopropanol for 40% in 83 mL | 33 mL |

BEFORE START INSTRUCTIONS

**Managing Expectations**

- Our specimen vetting methods will identify unsuitable specimens and help to avoid unnecessary destructive sampling of unsuitable specimens. However, due to variation in preservation practices, we cannot completely guarantee that specimens identified as suitable will yield sequenceable DNA.
- We have successfully used these methods on liver, muscle, ovary and testis tissues as well as whole fish larvae and for samples as old as 1905.
- The DNA you extract from these specimens will be highly fragmented. In our experience, the mean fragment length is between 50 & 150bp.

**Sequencing Considerations**

- Amplicon-based sequencing approaches must be designed to target fragments in this range.
- *De novo* genome assembly from these sorts of samples is not recommended and care must be taken to select an appropriate reference genome for mapping. We have had success mapping of species to a reference of the same Genus, however, success will vary depending on evolutionary distance and reference quality.
- For library preparation, we have found that kits designed to take fragmented input work very well. For example, the IDT xGen Cell Free and FFPE kit.
- We highly recommend using a trimming-free read alignment approach such as kalign as implemented in the ngskit4b toolkit. See our manuscript in [Molecular Ecology Resources] for further details.
- Capture-based sequencing methods have been successfully applied to formalin-preserved specimens and we encourage use of our extraction protocol as input for capture approaches.

## Specimen vetting

57m

1 Visually assess the specimen. If the specimen appears decomposed, consider selecting an alternative specimen. Generally, specimens with discernible internal organs tend to have higher likelihood of success. If the internal organs have been removed, this may indicate that the specimen had begun to decompose prior to fixation and will be unsuitable for DNA sequencing. Consult with your curatorial team as, in some cases, removal of the internal organs may have been conducted for alternative purposes that may not have influenced DNA preservation. 15m

Safety information

Note: Treat all specimen media as though it contains formaldehyde. Use appropriate PPE and follow Safe Work Instructions for your facility.

2 From the jar containing your visually suitable specimen, take an aliquot of the specimen media into a 15 mL tube for down-stream measurement of residual formaldehyde and pH. If using a pH 1m

meter, you'll want to remove enough media such that you can adequately immerse the probe - typically at least 7 mL.

3 Measure the pH of the media. 20m

Note

We use an Orion Versa Star Pro benchtop pH meter (Thermo Scientific) but other meters that work in media containing ethanol may be suitable.

4 Measure the residual formaldehyde concentration [F] of the media. 20m

Note

We use MQuant test strips (Merck) and dilute the media in increments of 1:10 with ultra-pure water if [F] exceeds the upper detection limit of the test strips.

5 Rate your visually well-preserved specimen for suitability against your sequencing objectives. 1m

- If the pH measures lower than 6 or the [F] measures greater than 10,000 mg/L, consider using an alternative specimen or limit your interrogation to small amplicon-based approaches.
- If the pH is neutral and the [F] sits above 0 and below 10,000 mg/L, treat the specimen as formalin-fixed and well-preserved, thus suitable for broader-scale DNA sequencing, depending on extraction yield.
- If the pH is neutral and there is no detectable formaldehyde, treat the specimen as ethanol-preserved.

6 Dissect at least 50 mg of your target tissue and remove to a 2 mL tubes containing 70% ethanol.

Note

We have extensively tested this method with liver (largely due to the ease of sampling) as well as on smaller scales with muscle, ovary, testis and whole fish larvae with high success but other tissues may also be very suitable.

7 Transfer and store the dissected tissue at room temperature until further processing.

## Day 1 - Tissue Preparation

17h 20m

8 Pulverisation

Note

Prior to starting, ensure that you have stock of the following on hand chilled to 4°C:

50% and 30% ethanol
MilliQ water
Glycine Tris EDTA Buffer (GTE: 100mM glycine, 10mM Tris-HCl, pH 8.0, 1mM EDTA)

8.1 Weigh and record the total tissue mass, subsampling to 50 mg if more tissue is available. Note: More than 50 mg can be processed. Here we have limited it to 50 mg as we find this amount pulverises well without rupturing the tissueTUBE bag. 10m

8.2 Transfer the tissue to an extra thick TT05 Covaris tissueTUBE bag and attach a 1 mL Covaris milliTUBE. 1m

Note

Both the TT05 and TT1 tissueTUBEs work well in this application and either can be selected depending on the adapters you have available. In either case, we recommend using the extra thick versions. Here, we use the milliTUBE to seal the bag and serve as a point to hold the tissueTUBE in place with the appropriate insertion tool during pulverisation. If you opt to use a TT1 paired with a 1 mL milliTUBE, you will also need to use an adapter. Alternatively, you can use a tissueTUBE plug to seal the bag and use an appropriate tissueTUBE holder to hold the bag in place during pulverisation.

Note

If you do not have access to a CryoPrep, alternative methods of pulverisation may be suitable. We find that very fine pulverisation as is achieved with a TissueLyzer is not necessary or advantageous. Whichever method you choose, aim to pulverise under cryogenic conditions to the consistency of rough sand.

8.3 Dispense ~ 1L liquid nitrogen to a cryo dewar and flash freeze the tissueTUBE bag through immersion for approximately 30 sec. 5m

Safety information

Wear appropriate cryogenic PPE and follow your facility's Safe Work Instructions for working with liquid nitrogen.

8.4 Apply 2-3 level 6 impacts to the tissueTUBE with the Covaris CryoPrep, flash freezing the sample between impacts. 5m

Note

Check for damage to the bag between impacts and do not apply further impacts if the bag is loosing integrity.

Expected result

The material will appear flattened rather than pulverised to a fine powder.

9 Tissue re-hydration and formaldehyde mop-up

9.1 Resuspend the tissue in cold 50% ETOH and transfer to a 15 mL tube using a wide-bore pipet tip. Rinse the bag with cold 50% ETOH to collect all material. Bring volume to 10 mL with cold 50% ETOH. Rock for 10 min at 4°C. 20m

9.2 Spin 10min at 750 x g 4°C. Remove supernatant and resuspend tissue in 10 mL cold 30% ETOH. Rock for 10 min at 4°C. 20m

Note

You want the material to pellet such that you can easily remove the supernatant but not so much that it is difficult to resuspend. This can be achieved at 750 x g but you can increase the centrifugation force if the material hasn't pelleted sufficiently.

9.3 Spin 10min at 750 x g 4°C. Remove supernatant and resuspend tissue in 10 mL cold milliQ water. Rock for 10 min at 4°C. 20m

9.4 Spin 10min at 750 x g 4°C. Remove supernatant and resuspend tissue in 10 mL cold GTE buffer. Rock over night at 4°C. 16h

## Day 2 - Hot Alkaline Lysis

50m

10 Spin the tube for 10 min at 750 x g 4°C. Remove and discard all but 1 mL of the GTE buffer. 10m

11 Resuspend the tissue in the 1 mL of retain GTE and transfer the tissue to a 2 mL screw-cap tube with a wide-bore pipet. 5m

12 Spin the screw-cap tube for 10 min at 750 x g 4°C. Remove the supernatant and add 500 µL Alkaline Lysis Buffer (0.1NaOH, pH 13; 1% SDS) to the pellet. 10m

Safety information

Seek and follow Safety Data Sheets for NaOH and SDS.

13 Secure the tube upright in an autoclave save container and autoclave at 120 °C for 25 minutes. 25m

Note

We use a tip box to contain the tubes during autoclaving and prop the tubes up with aluminium foil. If your autoclave does not have a 25 minute setting, it is sufficient to run multiple shorter cycles to achieve at least 25 minutes "hot time".

Expected result

If lysis is complete, the tube should be relatively clear without chunks of tissue remaining. The colour of the lysate typically ranges from completely clear to dark red-brown.

## Day 2 - DNA Purification

2h 15m

14 Phenol:chloroform:isoamyl alcohol extraction

Safety information

Seek and follow Safety Data Sheets for phenol:chloroform:isoamyl alcohol

14.1 Add equal volume phenol:chloroform:isoamyl alcohol (25:24:1) to the screw-cap tube of lysate, briefly vortex to resuspend and then rock for 5 min at room temperature. 7m

14.2 Centrifuge the tube at maximum speed in a microcentrifuge for 5 min at room temperature. 5m

14.3 Remove the aqueous phase to a new 2 mL tube and add 100 µL 10 mM Tris-HCl pH 8.0 to the organic phase. Rock the tube containing the organic phase for an additional 5 min. 7m

Note

Adding the Tris-HCl here is called a back-extraction and helps to improve recovery.

14.4 Centrifuge the organic phase tube at maximum speed in a microcentrifuge for 5 min at room temperature. Combine the aqueous phase from this extraction with that recovered from the first extraction. Discard organic phases in the appropriate waste stream. 7m

14.5 OPTIONAL: Add 1 µL RNase A to the extract and incubate at room temperature for 30 min. 30m

14.6 Repeat the phenol:chloroform:isoamyl alcohol extraction.

**Note**

If at any point the aqueous phase is cloudy, add 200 µL 10 mM Tris-HCl, pH 8.0 and perform the extraction again. Repeat the phenol:chloroform:isoamyl alcohol extraction at least one or two more times or until the aqueous phase is clear and can be cleanly removed. The aqueous phase may be very dark brown in colour but shouldn't be cloudy, which indicates the presence of protein.

15 Chloroform clean-up

15.1 Add equal volume chloroform and rock for 5 min at room temperature. 7m

15.2 Centrifuge the tube at maximum speed in a microcentrifuge for 5 min at room temperature. Remove the aqueous phase to a new 2 mL tube. 7m

15.3 Repeat chloroform extraction until the phenol is completely removed. Usually, we only perform one chloroform extraction and trust that the phenol is removed if we were able to remove the aqueous phase cleanly.

16 DNA concentration with SPRI beads

16.1 To each tube, add 1.5X volume small fragment-optimised bead solution and incubate at room temperature for 15 minutes. 17m

16.2 Briefly spin the tubes and place them on a magnet for 10 minutes or until the solution is clear. 10m

**Note**

The solution may still appear brown at this point if the aqueous phase was brown to begin with.

16.3 Remove the supernatant and wash the bead pellet twice with fresh 70% ethanol. 5m

16.4 Briefly spin the tubes and return to the magnet. Remove remaining ethanol with a small volume pipette and allow the beads to dry for 30 seconds. 1m

16.5 Add 30 µL 10 mM Tris-HCl, pH 8.0 and resuspend the beads by pipetting up and down. 1m

16.6 Incubate the tube at 37°C for 15 minutes then pellet the beads on the magnet once again for 15 minutes. 30m

16.7 Transfer the supernatant containing your extracted DNA to a new tube. 1m

## Day 2 - DNA QC

17 Measure the DNA concetration via Qubit and/or Tapestation.

Note

The quantity and fragment sizes we achieve are generally within the range of the Qubit dsDNA HS kit and Tapestation High Sensitivity D1000 kits.

Expected result

Samples yielding ≥200 ng DNA/50 mg tissue with mean fragment sizes above 150 bp are generally suitable for sequencing.
