# Supplementary figures and images for: Hot alkaline lysis gDNA extraction from formalin-fixed archival tissues
Source: PLoS One. 2024 Jan 2;19(1):e0296491. doi: 10.1371/journal.pone.0296491 (PMC10760679; doi:10.1371/journal.pone.0296491)

Figure 1B - Generated with Tapestation D1000 ScreenTape

Filename: 14390.D1000

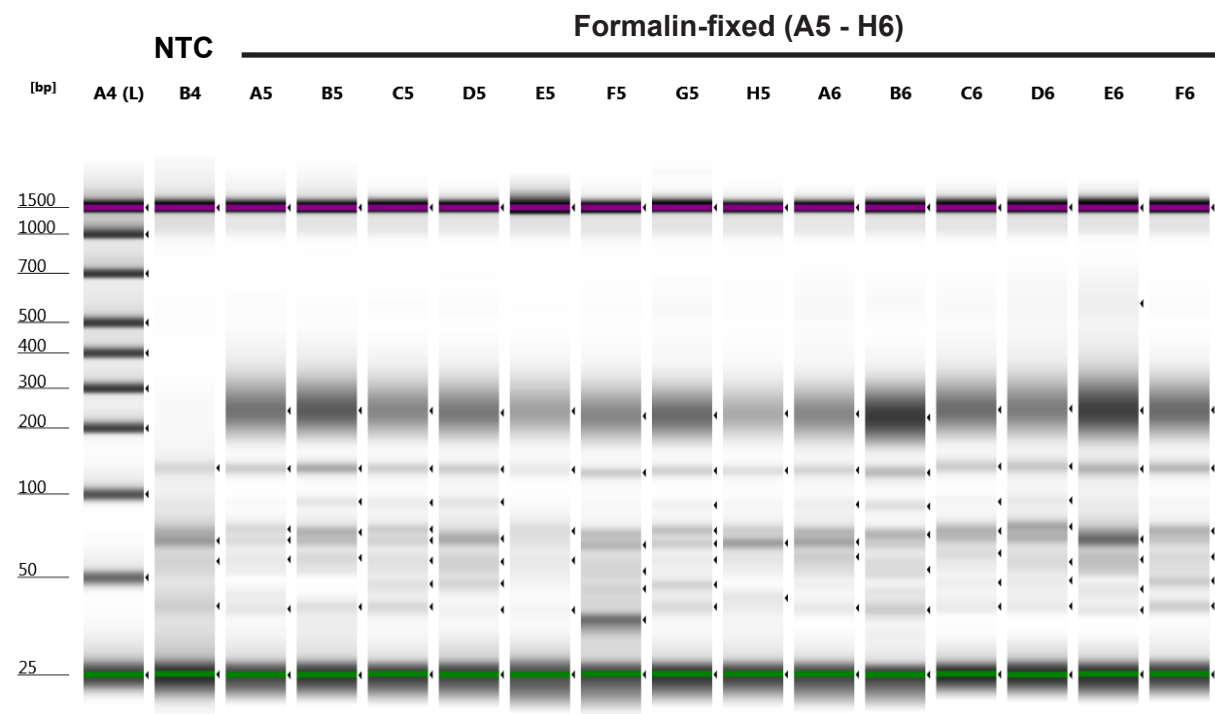

Default image (Contrast 100%)

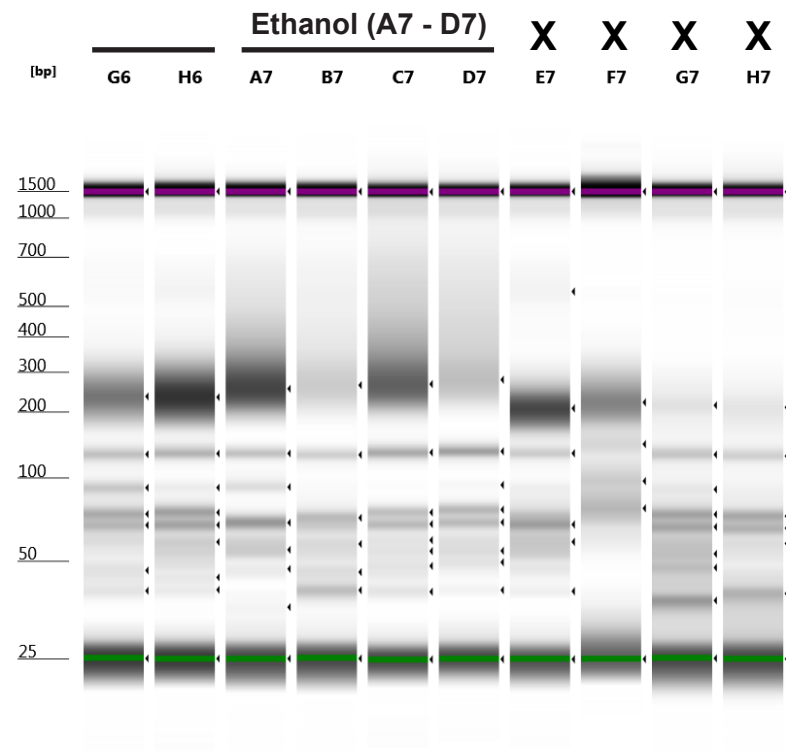

Supplement: S1 Raw images — (PDF) [file pone.0296491.s002.pdf]
