# Supplementary material for: Hot alkaline lysis gDNA extraction from formalin-fixed archival tissues
Source: PLoS One. 2024 Jan 2;19(1):e0296491. doi: 10.1371/journal.pone.0296491 (PMC10760679; doi:10.1371/journal.pone.0296491)
Supplement: Attachment — Submitted filename: ResponseToReviewers.pdf [file pone.0296491.s003.pdf]

Australian National Wildlife Collection | CSIRO
Building 17 ANWC, 80 Bellenden Street, Crace, ACT 2911

csiro.au | ABN 41 687 119 230

7 December 2023

Aldo Corriero, Ph.D.
Academic Editor
PLOS ONE

Dear Dr Corriero,

Here we provide responses to each point raised by yourself and Dr Imran.

Journal requirements:

1. Please ensure that your manuscript meets PLOS ONE's style requirements, including those for file naming.

We have made a few minor changes throughout to adhere to the style requirements, including removing the post codes from the author affiliations.

2. We note that you have provided funding information that is currently declared in your Funding Statement. However, funding information should not appear in the Acknowledgments section or other areas of your manuscript. We will only publish funding information present in the Funding Statement section of the online submission form.

Please remove any funding-related text from the manuscript and let us know how you would like to update your Funding Statement.

We have removed funding details from the Acknowledgments Section.

3. PLOS ONE now requires that authors provide the original uncropped and unadjusted images underlying all blot or gel results reported in a submission's figures or Supporting Information files.

We have included the requested file as S1_raw_images.pdf.

4. We note that Figures 1A and 1B in your submission contain copyrighted images. We require you to either (1) present written permission from the copyright holder to publish these figures specifically under the CC BY 4.0 license, or (2) remove the figures from your submission.

All images in both figures were created by the authors.

5. Please review your reference list to ensure that it is complete and correct.

No changes have been made to the reference list.

Response to Reviewers' comments:

Minor comments:
1. In my point of view, it will be better if the authors discuss about the further improvement of the protocol keeping in mind its expected limitations such as decrease in DNA quantity and/or fragment size with every step of purification and its impact on genetic information obtained.

We have added a paragraph and a half addressing these points to the Expected Results section (now lines 149-167).

2. Line 58: Kindly remove (Straube) (6)

Here, we removed "(Straube)" from the text (now line 61).

3. Line 72: Is it necessary to have the word "cover" in "recover up to 25X whole genome cover from"

Changed to "yield" (now line 75).

4. Line 144: Kindly change "reverse (read)" to "reverse (red)"

Amended (now line 150).

5. Line 147: Kindly change "Due to the high degree of DNA fragmentation" to "Due to high degree of DNA fragmentation"

This line was edited to remove this text with the addition of the final paragraph (now line 173).

6. Line 159 – 162: Is it OK to write down the below sentences for Supporting information as such?
The protocol in PDF format available from protocols.io must be provided as Supporting Information file 1, with the caption: S1: Step-by-step protocol, also available on protocols.io

Amended (now line 183).

Sincerely,

Dr. Erin Hahn
Research Scientist
Australian National Wildlife Collection
CSIRO National Collections and Marine Infrastructure

erin.hahn@csiro.au
+61 262 421 544
